# Immunohistochemical “null” pattern of all four mismatch repair proteins in Lynch syndrome–associated small bowel adenocarcinoma: a case report and comprehensive genomic profiling

**DOI:** 10.1007/s00428-025-04232-0

**Published:** 2025-09-02

**Authors:** Satoko Kageyama, Masayuki Ota, Takanori Aihara, Kaito Nakama, Jun-ichiro Ikeda

**Affiliations:** 1 Department of Diagnostic Pathology, Graduate School of Medicine, Chiba University, 1-8-1 Inohana, Chuo-Ku, Chiba, Japan; 2 Department of Pathology, Chiba University Hospital, 1-8-1 Inohana, Chuo-Ku, Chiba, Japan

**Keywords:** Mismatch repair protein, Small bowel adenocarcinoma, Lynch syndrome, Jejunum, Immunohistochemistry, Null-phenotype

## Abstract

Immunohistochemistry for mismatch repair (MMR) proteins is important for evaluating the molecular background of MMR-deficient tumors, including those with Lynch syndrome (LS). The four major MMR proteins function as heterodimers (MLH1/PMS2 and MSH2/MSH6), and usually only one of the MMR subsystems is impaired. However, rare cases of concurrent immunohistochemical loss of all four MMR proteins, termed “null” phenotype, have been reported. We present the first reported case of small bowel adenocarcinoma (SBA) showing a null-phenotype. A 62-year-old man developed jejunal cancer. Immunohistochemical analysis showed complete loss of all four MMR proteins in the entire tumor. Genetic testing revealed LS with a germline pathogenic variant in *MSH2*, and somatic mutations in *MSH2* and *MLH1* were also detected. No *BRAF* V600E mutation was found. Combined with comprehensive cancer genomic profiling, this report provides insight into an exceptional MMR-deficient tumor and SBA, a relatively rare disease given its surface area.

## Introduction

The mismatch repair (MMR) gene system is a cellular mechanism that detects and corrects DNA replication errors [1]. DNA mismatch repair deficiency (dMMR) causes microsatellite instability (MSI), which is related to oncogenesis [1, 2]. The main MMR proteins are mutL homologue 1 (MLH1), mutS homologue 2 (MSH2), mutS homologue 6 (MSH6), and postmeiotic segregation increased 2 (PMS2). Pathogenic germline variants in these genes are the principal cause of Lynch syndrome (LS), an autosomal dominant inherited disease that increases the risk of malignant tumors in various organs [1]. MLH1/PMS2 and MSH2/MSH6 function as heterodimers, with PMS2 and MSH6 becoming unstable and displaying negative immunohistochemical (IHC) staining in the absence of MLH1 and MSH2, respectively [2]. Only one DNA MMR subsystem typically loses function in dMMR malignancies [2]. However, rare cases of concurrent IHC staining loss for all four MMR proteins, termed “null” phenotype, have been reported [3–11]. The epidemiology and clinicopathological significance of such cases remain unclear. To the best of our knowledge, reports of the null MMR staining phenotype have been limited to colorectal and gastric cancers [3–11], and there have been no similar reports of small bowel adenocarcinoma (SBA). Herein, we present a novel case of primary jejunal carcinoma in a 62-year-old male diagnosed with LS who exhibited a null phenotype IHC pattern for MMR proteins.

## Case report

A 62-year-old man with a history of surgically resected rectal mucinous adenocarcinoma (pT3N1M0) and ascending colon adenocarcinoma not otherwise specified (NOS) (pT1bN0M0) at the age of 51 years presented with abdominal pain and vomiting. Computed tomography (CT) image showed a mass lesion at the proximal jejunum, which was diagnosed as adenocarcinoma by biopsy. The patient subsequently underwent partial jejunectomy with lymph node dissection at our hospital.

The patient’s family history was significant for LS. His sister had been diagnosed with multiple colorectal cancers and uterine corpus cancer and was confirmed to have LS. His brother died of a brain tumor at 16 years old. His mother had a history of colorectal cancer and cervical cancer and was alive at 90 years old at the time of this report. His father had a history of gastric cancer at 55 years old and died at 62 years old.

**Fig. 1:**
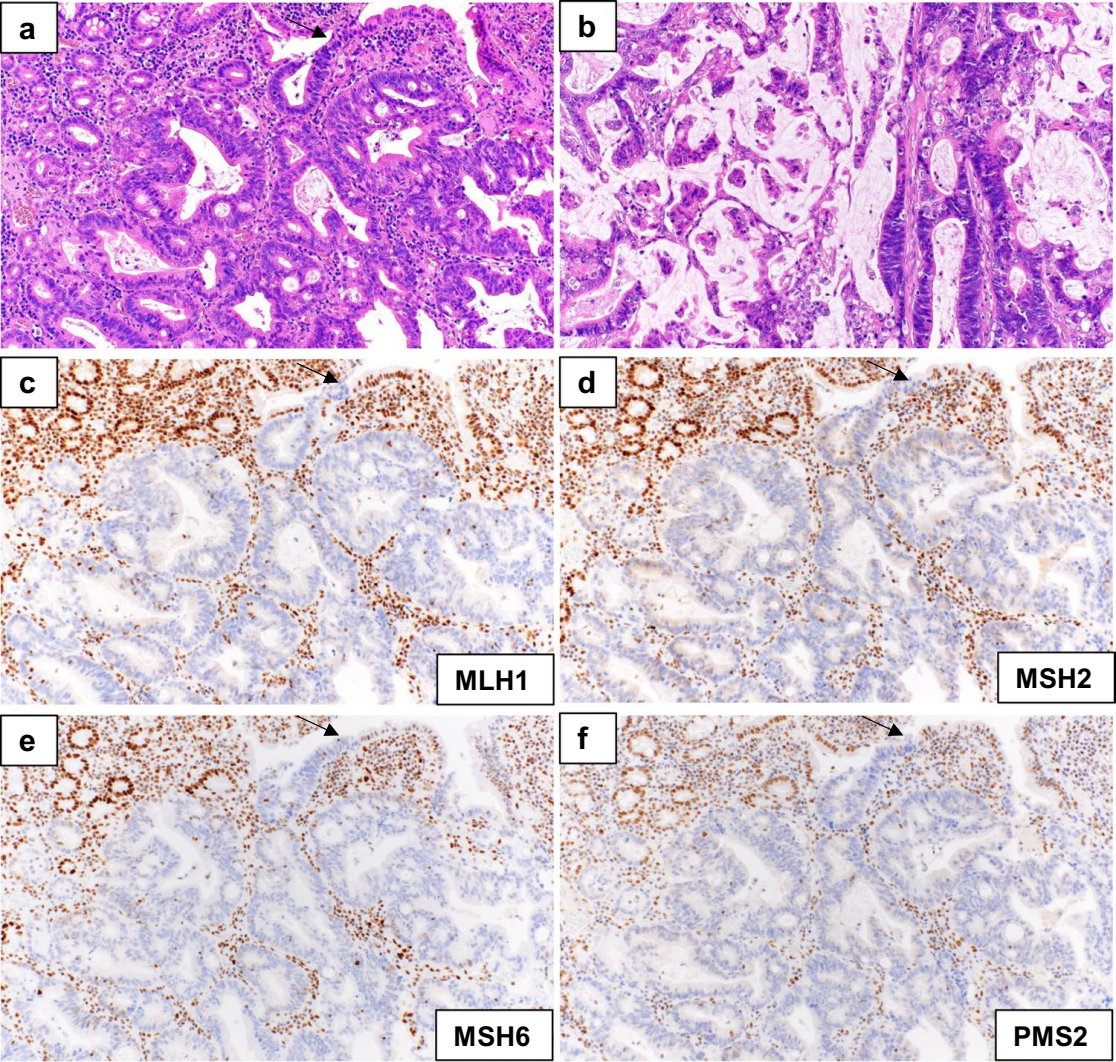
Histopathological images of jejunal cancer. **a**, **b** Hematoxylin and eosin stain images. **a** Adenocarcinoma NOS. A black arrow points to the intraepithelial lesion. **b** Mucinous carcinoma component. **c**–**f** Immunohistochemistry. MLH1 (**c**), MSH2 (**d**), MSH6 (**e**), and PMS2 (**f**) are all deficient. Black arrows point to the intraepithelial lesion

Histopathological examination of the jejunal specimen revealed adenocarcinoma NOS with focal mucinous adenocarcinoma component (Fig. 1a, b). The presence of an intraepithelial lesion indicated a primary tumor. Venous and lymphatic invasion were identified. The radial margin was positive, while the proximal and distal margins were negative. According to UICC 8th edition, the tumor was staged at least pT3, pN2 (4/5), and cM0 (stage IIIB). The tumor showed moderate tumor-infiltrating lymphocytes.

Immunohistochemical analysis was performed on 4-μm-thick formalin-fixed paraffin-embedded tissue sections using an automated immunostaining system (Dako Autostainer Link 48, Agilent Technologies, Santa Clara, CA, USA) with the following antibodies: MLH1 (clone M1, prediluted, Roche Diagnostics, Rotkreuz, Switzerland), MSH2 (clone FE11, prediluted, Agilent Technologies), MSH6 (clone EP49, prediluted, Agilent Technologies), and PMS2 (clone EP51, prediluted, Agilent Technologies). A single antibody was used for each MMR protein, and the loss of each protein was confirmed independently. Both external positive controls and internal positive controls (normal epithelial cells and inflammatory cells within the same slide) demonstrated adequate staining for all four MMR proteins. The comprehensive evaluation of all four staining results demonstrated complete loss of all four MMR proteins in the jejunal cancer (Fig. 1c–f), constituting a “null” phenotype. On the other hand, the patient’s previous rectal and ascending colon cancers showed positive staining for MLH1 and PMS2, with negative staining for MSH2 and MSH6.

FoundationOne CDx (Foundation Medicine, Cambridge, MA, USA) test of the jejunal cancer specimen detected three significant variants: *MSH2* F836fs*5 (variant allele frequency, VAF 47.4%), *MSH2* D716fs*4 (VAF 12.0%), and *MLH1* R659* (VAF 13.2%). The tumor demonstrated MSI-high and high tumor mutation burden (43 mutation/megabase). Tumor cell ratio estimated by the histopathological image was 20% and calculated by next-generation sequencing was 23.4%. *MSH2* F836fs*5 had a VAF of approximately half of all alleles, consistent with a germline variant. The other variants, *MSH2* D716fs*4 and *MLH1* R659*, accounted for nearly half the proportion of tumor cells, consistent with somatic variants. A peripheral blood test confirmed *MSH2* F836fs*5 as a germline variant, which was also found in the patient’s sister. Genetic testing for the *MSH2* F836fs*5 variant was not performed in the parents: the father had already died at the time of diagnosis of LS in the patient and his sister, and genetic testing was not recommended for the mother by her attending physician despite her cancer history. Other genomic findings were *KRAS* wildtype, *NRAS* wild type, *ARID1A* P1326fs*155, *ARID1A* S617fs*2, *ASXL1* G646fs*12, *BCORL1* P1681fs*20, *CD79A* R131fs*61, *CTCF* T204fs*18, *EED* R133W, *FBXW7* R465C, *JAK1* P430fs*2, *KMT2D(MLL2)* R2801*, *NSD (WHSC1 or MMSET)* E1344fs*91, *PIK3CA* H1047R, *PPP2R1A* R105Q, *RNF43* R117fs*41, *RNF43* G659fs*41, and *SETD2* T305fs*4.

The patient received postoperative immunotherapy with pembrolizumab. Seven months after surgery, he required an operation for intestinal obstruction. Although the tumor was under control, the patient died 23 months after surgery, presumably from unrelated causes.

## Discussion

In this report, we illustrated an unusual immunostaining pattern in primary jejunal cancer, with the loss of all four MMR proteins. There have been only a limited number of reported cases in which all four of these proteins were negative, called “null” immunophenotype [3–11]. Our case presented the first instance of a null IHC phenotype in primary SBA and the third in confirmed LS cases, following two cases of colon cancer [3, 4]. To our knowledge, detailed information was available on only 10 cases showing such an abnormal IHC pattern [3–9], including nine colorectal cancers and one gastric cancer (Table 1). Although there was information on four MMR-negative cases, detailed information and discussion on the individual patients were unavailable. Therefore, five of the 580 Asian/Korean gastric cancer cases [10] and seven of the 100 Pakistani colorectal cancer cases [11] were not included in Table 1.

**Table 1:** Summary of clinical and genetic findings of mismatch repair null phenotype cases

Case [ref. number]	Cancer type	Age	Sex	Germline variant of MMR gene	Somatic variant of MMR gene	*BRAF* V600E	*MLH1*-PM	LS/sporadic
[3]	CRC	63	F	*MSH2* c.(2634 + 1_2635-1)_(*279 + 1-?)del (p.(?))	NA	(-)	(+)	LS
[4]	CRC	63	F	***MSH2* c.G1759C (p.G587R)**	NA	(-)	(+)	LS
[5]	CRC	80	F	(-)	*MSH2* c.1861 C>T (p.R621*) *MSH2* c.633dupG (p.K212Efs*20) *MSH2* c.298G>A (p.V100I)	(+)	(+)	Sporadic
[6]	CRC	70	M	(-)	NA	NA	(-)	Sporadic
[7]	CRC	78	F	NA	NA	(+)	(+)	Sporadic
[7]	CRC	81	F	NA	NA	(+)	(+)	Sporadic
[7]	CRC	83	F	NA	NA	(+)	(+)	Sporadic
[7]	CRC	76	M	NA	NA	(+)	(+)	Sporadic
[8]	CRC	75	F	(-)	*MSH6* c.NA (p.F1088Sfs2*)	(+)	(+)	Sporadic
[9]	GC	67	M	(-)	***MSH2* a missing copy number in exon 14** *MLH1* c.1039-13_1039-8del *PMS2* c.1799T>C (p.M600T) *MSH6* c.2693C>A (p.P898H)	NA	(+)	Sporadic
Our case	SBA	62	M	*MSH2* c.2507delT (p.F836fs*5)	*MSH2* c.2146delG (p.D716fs*4) *MLH1* R659*	(-)	Not examined	LS

Gene mutations considered to be pathogenic are shown in bold. Abbreviations: *Ref.* reference, *MMR* mismatch repair, *MLH1-PM*, *MLH1* promoter methylation,  *LS* Lynch syndrome, *CRC *colorectal cancer, *F* female, *NA* not available, *M* male, *GC* gastric cancer, *SBA* small bowel adenocarcinoma

Of the 11 cases in Table 1, three cases, including ours, were LS-related, and eight were confirmed or suspected to be sporadic. All three LS cases had germline mutations in *MSH2* but no *BRAF* mutations. The two previously reported cases of colorectal cancer had methylation of the *MLH1* promoter region [3, 4], and it is possible that the same phenomenon occurred in our case. In sporadic cases, seven of eight cases had *MLH1* promoter methylation, and six of those seven had *BRAF* V600E (Table 1). Notably, in three sporadic cases, MLH1 and PMS2 were negative in the entire tumor, but MSH2 and MSH6 were negative only in a portion of the tumor [7]. This finding suggested that methylation of the *MLH1* promoter region by *BRAF* V600E was the cause of tumor development and that mutations in *MSH2* occurred after tumor development [7].

In our case, all four MMR proteins were lost in all tumor cells, including intraepithelial carcinoma components, and presumably, all tumor cells had somatic mutations in *MLH1* and *MSH2*. Therefore, it was more reasonable to assume that both MMR subsystems, MLH1/PMS2 and MSH2/MSH6, were impaired at the beginning of cancer development. This was an interesting finding, given that although the small bowel occupies 75% of the gastrointestinal tract length and 90% of the mucosal surface area, primary small bowel cancer accounts for only about 5% of gastrointestinal cancers [12, 13].

It is known that some cases of small bowel cancer have genetic alterations in *ARID1A* and *PIK3CA* [14]. In our case, *ARID1A* P1326fs*155, *ARID1A* S617fs*2, and *PIK3CA* H1047R were detected. Their % VAFs were 12.3%, 11.8%, and 12.4%, respectively, almost the same as somatic variants of *MLH1* and *MSH2*, corresponding to one allele in all tumor cells. Therefore, it is possible that *ARID1A* and *PIK3CA* mutations triggered SBA oncogenesis. It was possible that dysfunction of two MMR subsystems leads these mutations. Furthermore, in this case, many genetic mutations other than MMR genes and known small bowel cancer-related genes were also detected through comprehensive genome profiling.

In recent years, the clinical application of next-generation sequencing has expanded, and opportunities for comprehensive cancer genome profiling are increasing. However, reports of null phenotypes, including our case, suggest that combining IHC with genetic findings is important. Histopathological findings, protein properties and distribution in IHC, genetic mutations, and epigenetics should be integrated comprehensively. In cases of null phenotype, considering whether the mutation was secondary to *MLH1* or *MSH2* deletion or whether the loss of both MMR subsystems contributed to carcinogenesis will help elucidate the mechanism of carcinogenesis and stratify cases.

Additionally, our case raises an important consideration for current diagnostic practices. Recent studies have proposed that testing only PMS2 and MSH6 by IHC might be sufficient and cost-effective for screening dMMR in colorectal and other cancers [15]. This approach is based on the conventional understanding that PMS2 and MSH6 loss reflects dysfunction in their heterodimeric partners, MLH1 and MSH2. However, null phenotype cases like ours demonstrate that exceptional patterns exist that would be misinterpreted by such simplified testing strategies. Had our case been tested only for PMS2 and MSH6, the results would have correctly identified the dMMR status but potentially misattributed the underlying genetic cause, affecting genetic counseling and therapeutic decisions. This observation underscores the continued value of comprehensive MMR protein testing, particularly in cases with complex clinical presentations or strong family histories of cancer.

## Conclusion

We have presented a case of SBA in a patient with LS that showed exceptional MMR immunostaining results with all four MMR proteins being negative (null phenotype). To deepen our understanding of dMMR malignancies and primary SBA, further accumulation of such characteristic cases is necessary, integrating histopathology, immunohistochemistry, and comprehensive genetic testing. The integrated approach will enable better analysis of the incidence, treatment response, and prognosis of tumors exhibiting such rare immunophenotypes, ultimately improving diagnostic accuracy and treatment strategies for patients with complex presentations of dMMR cancers.
